# Oral colonization by gram-negative bacilli in patients with hematologic malignancies and solid tumors compared with healthy controls

**DOI:** 10.1186/s12903-023-03172-y

**Published:** 2023-07-08

**Authors:** Karla E. Santibañez-Bedolla, Maria J. Orozco-Uriarte, Jose A. Alvarez-Canales, Alejandro E. Macias, Lauro F. Amador-Medina

**Affiliations:** 1 Bajio Regional High Specialty Hospital, San Carlos La Roncha C.P. 37544, Leon Guanajuato, Mexico; 2 Department of Medicine, University of Guanajuato, San Carlos La Roncha C.P. 37660, Leon Guanajuato, Mexico

**Keywords:** Mouth, Oral colonization, Gram-negative bacilli

## Abstract

**Background:**

Colonization of the oropharynx with gram-negative bacilli (GNB) is considered a negative prognostic factor in immunocompromised individuals. Hemato-oncologic patients represent a high-risk group due to their immunodeficiencies and associated treatments. This study aimed to determine the rates of oral colonization by GNB, associated factors, and clinical outcomes in patients with hematologic malignancies and solid tumors compared with healthy subjects.

**Methods:**

We conducted a comparative study of hemato-oncologic patients and healthy subjects from August to October 2022. Swabs were taken from the oral cavity; specimens with GNB were identified and tested for antimicrobial susceptibility.

**Results:**

We included 206 participants (103 hemato-oncologic patients and 103 healthy subjects). Hemato-oncologic patients had higher rates of oral colonization by GNB (34% vs. 17%, P = 0.007) and GNB resistant to third-generation cephalosporins (11.6% vs. 0%, P < 0.001) compared to healthy subjects. *Klebsiella* spp. was the predominant genus in both groups. The factor associated with oral colonization by GNB was a Charlson index ≥ 3, while ≥ 3 dental visits per year were a protective factor. Regarding colonization by resistant GNB in oncology patients, antibiotic therapy and a Charlson index ≥ 5 were identified as associated factors, while better physical functionality (ECOG ≤ 2) was associated with less colonization. Hemato-oncologic patients colonized with GNB had more 30-day infectious complications (30.5% vs. 2.9%, P = 0.0001) than non-colonized patients.

**Conclusion:**

Oral colonization by GNB and resistant GNB are prevalent in cancer patients, especially those with higher scores on the severity scales. Infectious complications occurred more frequently in colonized patients. There is a knowledge gap about dental hygiene practices in hemato-oncologic patients colonized by GNB. Our results suggest that patients’ hygienic-dietary habits, especially frequent dental visits, are a protective factor against colonization.

**Supplementary Information:**

The online version contains supplementary material available at 10.1186/s12903-023-03172-y.

## Background

The oral cavity microbiota consists mainly of anaerobic bacteria and vaguely classified streptococci known as the viridans group; however, this composition varies from person to person [1]. Aerobic Gram-negative bacilli (GNB) and facultative anaerobes are not considered members of the oropharyngeal microbiota [2]. The presence of GNB in the oral cavity of healthy subjects is transient due to the efficacy of oral mechanisms for their eradication, such as saliva pH, enzymes, and immunoglobulin content. The GNB, such as *Klebsiella* species and other *Enterobacteriaceae* (*Enterobacter* spp. and *Serratia* spp.), can occasionally be found in healthy oral microbiota [3]. These organisms can cause systemic infection through three main mechanisms: dissemination into the bloodstream from the periodontal environment, bronchoaspiration, and migration to the gut microbiota [4]. In cancer patients, the GNB is associated with an increased risk of infectious complications, particularly neutropenic fever, and bacteremia [5, 6].

The recent emergence of organisms resistant to multiple antibiotics contributes to GNB-associated infections being one of the most common causes of death in patients with hematologic malignancies [7]. Species such as *Escherichia coli* and non-fermenting bacilli (*Pseudomonas* spp. and *Acinetobacter* spp.) are considered relevant as they are frequently isolated multidrug-resistant (MDR) GNB in hospitalized patients and are associated with an increased risk of secondary infections [8].

Identification of oral colonization by GNB in cancer patients could allow for the appropriate selection of empiric antibiotic regimens and improve the response to infections caused by MDR organisms [9]. Therefore, the present study aimed to determine the rates and factors associated with GNB oral colonization in patients with hematologic malignancies and solid tumors, compared with healthy subjects, as well as evaluating the clinical outcomes in the patients’ group.

## Materials and methods

### Study design and participants

We performed a cross-sectional, analytical study with a nested cohort at the Bajio Regional High Specialty Hospital, between August and October 2022. This center is a tertiary care hospital focused on the care of hemato-oncology and immunosuppressed patients. The study was conducted in accordance with the Declaration of Helsinki and the Strengthening the Reporting of Observational Studies in Epidemiology guidelines (STROBE) [10]. It was approved by the ethics and research committee of the hospital (registration number CEI-005-2022 and CI/HRAEB/017/2022, respectively) and all participants provided informed consent.

A total of 206 subjects were included in the study, of whom 103 were patients (ambulatory or hospitalized) diagnosed with hematologic malignancies such as acute leukemias, lymphomas, or multiple myeloma and solid tumors. Baseline characteristics, clinical data, and recent antibiotic treatment were recorded in a database. The comparison group included healthy subjects randomly selected from the general population across all socioeconomic strata without the disease. All participants had to be ≥ 18 years of age. Exclusion criteria included antibiotic use within 15 days prior to the sample collection, the presence of chronic inflammatory systemic disease or autoimmune disease, and treatment with oncologic or immunosuppressive agents. The Charlson comorbidity index was used to predict mortality in all participants, while the Eastern Cooperative Oncology Group (ECOG) Performance Status Scale was used to assess physical performance in the patient group.

### Sampling

We collected the specimens from the oropharynx with sterile swabs, performing the procedure by rubbing the soft palate, buccal mucosa, and gingival junction for 20 s. In healthy subjects, swabbing was performed after at least eight hours of fasting, whereas samples from cancer patients were taken at any time during the day, with no restrictions on dental hygiene or fasting time. To evaluate oral hygiene, we analyzed the following variables: frequency of tooth brushing, flossing, and mouthwash use, presence of periodontal disease or caries, number of dental visits per year, and history of dental procedures or treatments.

### Microbiological methods

The specimens were processed in the microbiology laboratory of the University of Guanajuato, inoculating them over selective agar (MacConkey medium, BD® BBL® Mexico City, Mexico) and the same MacConkey agar added with 1.6 µg/mL of ceftazidime. Incubation was performed for 24–48 h at 35 ± 2 °C under an aerobic atmosphere. We performed standard microbiological and biochemical procedures to identify the isolated organisms. Antimicrobial susceptibility testing was conducted following the criteria of the Clinical and Laboratory Standards Institute [11]. Bacteria resistant to one or more agents from three or more antimicrobial classes were considered multidrug-resistant (MDR) [12]. We assessed the Extended-spectrum beta-lactamase (ESBL) production by the double-disk synergy test [13]. For the present study, all the GNB isolated were identified, although a subanalysis was performed for the following relevant bacteria only: *E. coli, Citrobacter* spp., *Pseudomonas* spp., *Acinetobacter* spp., and *Stenotrophomonas maltophilia*; these GNB were considered clinically relevant because they tend to be acquired in the hospital setting, often have intrinsic resistance mechanisms and infection with these bacteria is associated with a worse prognosis [14, 15]. On the other hand, *Klebsiella, Enterobacter, Serratia*, and *Proteus* species live in water, soil, and occasionally in food, and in many cases are part of the intestinal flora of humans and animals [16–19].

### Statistical analysis

The sample size was calculated using the formula of comparison of two proportions, considering an alpha risk of 0.05 and a beta risk of 0.2. Based on initial results from 20 pairs, we estimated a colonization proportion of 40% in the group of hemato-oncologic patients and 20% in the group of healthy subjects. This resulted in a minimum sample size of 79 subjects per group.

Categorical variables were expressed as frequencies and proportions. Quantitative variables were expressed as means and standard deviations (SD) or medians and interquartile ranges (IQR), according to the nature of data distribution. Continuous variables were compared with parametric (Student’s t-test), or nonparametric (Mann-Whitney U) tests based on the data distribution. The chi-square or Fisher’s exact probability tests were used for categorical variables, depending on the expected data value in the contingency table cells. We calculated the odds ratios (OR) and their corresponding 95% confidence intervals (95%CI) by univariate analysis. Logistic regression was performed to assess risk factors associated with GNB colonization. Potential predictor variables for inclusion in the model entry were identified based on the statistically significant features in the univariate analysis. We then used a backward stepwise selection of these variables to investigate independent risk factors associated with oral colonization, completing the process until only statistically significant variables were considered. Only the variables and results of the final model are shown. Statistical significance was set at a p-value < 0.05. Data analysis was performed with SPSS v.21 statistical software (IBM, Chicago, IL).

## Results

A total of 206 participants were included, 103 healthy volunteers and 103 patients with hematologic malignancies or solid tumors. Clinical and demographic characteristics and the hygienic-dietary variables of each study group appear in Table 1. The colonization rate of GNB was 34% among patients with hematologic malignancies or solid tumors, while it was 17% among healthy subjects (OR, 2.53; 95% CI 1.32–4.86; P = 0.007), these results are shown in Table 2.

**Table 1:** Demographic, clinical, and hygienic-dietary characteristics of the participants

Characteristic	Hemato-oncologic patients (N = 103) n (%)	Healthy subjects (N = 103) n (%)	P value
Sex			
Male	47 (45)	59 (57)	0.12 *
Female	56 (54)	44 (43)	
Age (years)_ Median (IQR)	48 (35–61)	45 (41–50)	0.019 ‡
BMI (Kg/m^2^) _ Mean (± SD)	26.2 (6.9)	26.1 (3.6)	0.885 §
Hospitalization	65 (63)	-	-
Outpatient	38 (36)	-	-
Diagnosis			
Acute leukemias	22 (21)	-	-
Lymphomas	20 (19)	-	-
Multiple Myeloma	12 (12)	-	-
Solid tumor	49 (48)	-	-
Antibiotic therapy	31 (30)	-	-
History of previous hospitalizations	70 (67)	-	-
Recent chemotherapy	54 (52)	-	-
Visits to the dentist/year.			
0 times	65 (63)	41 (39)	0.008 *
1 time	22 (21)	34 (33)	
2 times	6 (5)	13 (12)	
3 times	10 (9)	15 (14)	
Tooth brushing/day			
0 times	13 (12)	0	< 0.001 *
1 time	29 (28)	7 (6)	
2–3 times	35 (34)	58 (56)	
>3 times	26 (25)	38 (36)	
Flossing	9 (8)	43 (41)	< 0.001*
Mouthwash use	13 (12)	30 (29)	0.006 *
Oral disease	31 (30)	4 (3)	< 0.001 *
Type of Oral Disease			
No disease	76 (73)	99 (96)	< 0.001 †
Gingivitis	20 (19)	2 (2)	
Periodontal	3 (2)	1 (1)	
Aphthae	4 (3)	1 (1)	
Presence of caries	82 (79)	44 (42)	< 0.001 *
Dental treatment	19 (18)	25 (24)	0.396 *
Smoking	22 (21)	13 (12)	0.138 *

IQR: Interquartile range. SD: Standard deviation

* Chi-square

†Fisher’s exact test

‡Mann-Whitney U test

§ Student’s T-test

**Table 2:** Analysis of microbiological variables in patients with hematologic malignancies or solid tumors and healthy subjects

Characteristic	Hemato-oncologic patients (N = 103) n (%)	Healthy subjects (N = 103) n (%)	OR 95% CI	P value
Colonized by GNB	36 (34.9)	18 (17.5)	2.53 (1.32–4.86)	0.007 *
Clinically relevant GNB isolated ‡	15 (14.5)	5 (4.8)	3.34 (1.16–9.56)	0.03 *
GNB isolated				
* Klebsiella* spp.	18 (17.5)	8 (7.8)	2.51 (1.04–6.07)	0.05 *
* Enterobacter* spp.	3 (2.9)	5 (4.8)	0.58 (0.13–2.52)	0.72 †
* Citrobacter* spp.	5 (4.8)	1 (0.9)	5.2 (0.59–45.34)	0.21 †
* Escherichia coli*	5 (4.8)	1(0.9)	5.2 (0.59–45.34)	0.21 †
* Pseudomonas* spp.	3 (2.9)	1(0.9)	3.06 (0.31–29.91)	0.62 †
* Stenotrophomonas maltophilia*	1 (0.9)	2 (1.9)	0.49 (0.04–5.54)	1 †
* Acinetobacter* sp.	1 (0.9)	0	-	1 †
Colonized by resistant GNB	12 (11.6)	0	28.3 (1.65-484.33)	< 0.001 *
Type of colonizing-resistant bacteria ESBL	2 (1.9)	-	-	-
AmpC B-lactamase	2 (1.9)	-	-	-
MDR	4 (3.8)	-	-	-
MDR and ESBL	1 (0.9)	-	-	-
Resistant to 1 antimicrobial group	3 (2.9)	-	-	-

ESBL: extended spectrum β-lactamase; MDR: multidrug resistant; GNB: Gram-negative bacilli. OR: Odds ratio. CI: Confidence interval

* Chi-square

† Fisher’s exact test

‡ Clinically relevant GNB isolated: *Citrobacter* spp., *Escherichia coli, Pseudomonas* spp., *Stenotrophomonas maltophilia, Acinetobacter* spp.

### Microbiological results

*Klebsiella* spp. was predominant in both study groups (Hemato-oncologic patients’ group 17.5% [18/103] and healthy subjects’ group 7.8% [8/103]). The colonization rate of relevant *Enterobacteriaceae* and non-fermenting bacilli was 14.5% (15/103) in the group of hemato-oncologic patients and 4.8% (5/103) in the healthy subjects´ group (OR, 3. 34; 95% CI 1.16–9.56; P = 0.03). Of these organisms, *E. coli* and *Citrobacter* spp. (4.8%) were the most frequently isolated in the patients´ group, while *S. maltophilia* (1.9%) was the most frequent in the healthy subjects’ group. The microbiological distribution of isolated GNB is shown in Table 2.

Colonization by GNB resistant to third-generation cephalosporins was more frequent in the hemato-oncologic patients´ group compared to healthy individuals [12 (11.6%) vs. 0 (0%); OR, 28.3; 95% CI 1.65–484.33; P < 0.001]. Regarding resistance phenotypes, two ESBL-producing *Klebsiella* spp. and two organisms with AmpC beta-lactamase-producing phenotype (*Klebsiella* spp. and *Citrobacter* spp.) were found. The resistance profile identified four MDR organisms (one *E. coli*, one *Enterobacter* spp., and two *Pseudomonas* spp.), one multidrug-resistant ESBL-producing *E. coli* and three organisms resistant to third-generation cephalosporins.

### Factors associated with oral colonization by GNB

Regarding univariate analysis, the total participants were divided into GNB colonized and non-colonized. The relevant factors associated with a higher colonization rate were the presence of oncologic disease (OR, 2.53; 95% CI 1.32–4.86; P = 0.007), the presence of solid tumors (OR, 2.81; 95% CI 1.42–5.58; P = 0.004) and a Charlson scale ≥ 3 (OR, 3.27; 95% CI 1.32–8.08; P = 0.01). Otherwise, three or more dental visits per year and mouthwash use were associated with a lower colonization rate (OR, 0.21; 95% CI 0.04–0.94; P = 0.04 and OR, 0.38; 95% CI 0.15–0.98; P **=** 0.039, respectively). Table 3 shows the clinical characteristics, hygiene-dietary variables, and severity scales by group and their association with oral colonization by GNB.

**Table 3:** Analysis of microbiological and hygienic-dietary variables as factors associated with oral colonization by GNB.

Univariate analysis				
Characteristic	Colonized by GNB (N = 54) n (%)	No colonized by GNB (N = 152) n (%)	OR 95% CI	P value
Group				
Hemato-oncologic patients	36 (67)	67 (44)	2.53 (1.32–4.86)	0.007 *
Healthy subjects	18 (33)	85 (56)		
Diagnosis				
No disease	18 (33)	85 (56)	0.39 (0.20–0.75)	0.007*
Acute leukemias	6 (11)	16 (11)	1.06 (0.39–2.87)	0.88 *
Lymphomas	8 (15)	12 (8)	2.02 (0.78–5.27)	0.22 *
Multiple Myeloma	1 (2)	11 (7)	0.24 (0.03–1.91)	0.19 †
Solid tumor	21 (39)	28 (18)	2.81 (1.42–5.58)	0.004 *
Charlson				
Low risk (0–2)	31 (57)	121 (80)	0.34 (0.17–0.67)	0.002*
Intermediate risk (3–4)	11 (20)	11 (7)	3.27 (1.32–8.08)	0.01 *
High risk (≥ 5)	12 (23)	20 (13)	1.88 (0.85–4.17)	0.17*
Visits to the dentist/year				
0 times	34 (63)	72 (47)	1.88 (0.99–3.57)	0.07*
1 time	13 (24)	43 (28)	0.80 (0.39–1.64)	0.67*
2 times	5 (9)	14 (9)	1.00 (0.34–2.93)	1.0 †
≥ 3 times	2 (4)	23 (15)	0.21 (0.04–0.94)	0.04*
Tooth brushing/day				
0 times	6 (11)	7 (5)	2.58 (0.82–8.08)	0.10 †
1 time	12 (22)	24 (16)	1.52 (0.70–3.30)	0.38 *
2 times	23 (43)	70 (46)	0.86 (0.46–1.62)	0.77*
≥3 times	13 (24)	51 (33)	0.62 (0.30–1.27)	0.26*
Flossing	10 (18)	42 (27)	0.59 (0.27–1.28)	0.25 *
Mouthwash use	6 (11)	37 (24)	0.38 (0.15–0.98)	0.039 *
Oral disease	10 (18)	25 (16)	1.15 (0.51–2.59)	0.88 *
Presence of caries	36 (67)	90 (59)	1.37 (0.71–2.64)	0.42 *
Dental treatment	8 (15)	36 (23)	0.56 (0.24–1.29)	0.24 *
Smoking	8 (15)	27 (17)	0.80 (0.34–1.89)	0.77 *

GNB: Gram-negative bacilli. OR: Odds ratio. CI: Confidence interval

* Chi-square

† Fisher’s exact test

A logistic regression model was performed using only the variables that were found to be statistically significant in the univariate analysis. These variables included the subjects’ group (hemato-oncology patients or healthy subjects), diagnosis, Charlson comorbidity scale, dental visits, and mouthwash use. The model identified as an independent risk factor for GNB colonization, a higher number of comorbidities [Charlson scale ≥ 3] (OR, 3.24; 95% CI 1.27–8.25; P = 0.014). In contrast, having three or more dental visits per year (OR, 0.21; 95% CI 0.04–0.98; P = 0.048) was identified as a protective factor. The results of the model are presented in Table 4.

**Table 4:** Logistic regression model

	Coefficient β	Std. Error	Adjusted odds ratio (95% CI)	P value
≥ 3 Dental visits per year	-1.535	0.777	0.215 (0.047–0.987)	0.048
Charlson scale ≥ 3	1.176	0.477	3.240 (1.272–8.255)	0.014

GNB: Gram-negative bacilli. CI: Confidence interval

A subanalysis was conducted to evaluate factors associated with colonization by third-generation cephalosporin-resistant GNB. These organisms were only isolated in patients with hematologic malignancies and solid tumors. The group was divided into colonized and non-colonized by third-generation cephalosporin-resistant GNB. Twelve individuals were included in the first group and 24 in the second group. An ECOG ≤ 2 (OR, 0.045; 95% CI 0.005–0.416; P < 0.001) was identified as a factor associated with avoiding colonization by third-generation cephalosporin-resistant GNB. The main factors associated with colonization by resistant GNB included antibiotic therapy and a Charlson scale ≥ 5 (OR, 6; 95% CI 1.32–27.28; P = 0.028 and OR, 5.32; 95% CI 1.17–24.14; P = 0.031, respectively). Other variables, such as neutropenia and lymphocytopenia, were evaluated, but no statistical significance was found. The results of this analysis are shown in Supplementary Tables 1, Additional File 1.

### Clinical outcome

To evaluate the clinical outcome, a 30-day follow-up was conducted exclusively for the group of hemato-oncologic patients colonized and not colonized by GNB. Regarding infectious complications, two (2.9%) patients developed an infectious complication in the 67 non-colonized hemato-oncologic patients´ group; while 11 (30.5%) patients developed an infectious complication in the 36 colonized hemato-oncologic patients´ group, (OR, 14.3; 95% CI 2.95–69.12; P ≤ 0.001). Two cases of non-colonized patients who developed infectious complications involved gram-negative bacilli bacteremia. Of the 11 colonized hemato-oncologic patients who developed an infectious complication, three (27%) were infected with relevant GNB (*Citrobacter* spp., *E. coli*, and *Pseudomonas* spp.), and five (45.5%) were colonized with GNB resistant to one or more antimicrobial agents. Of the total infectious complications developed, 36.4% were bloodstream infections (4/11), 9% were urinary tract infections (1/11), 27.2% were cases of pneumonia (3/11), and 27.2% were cases of neutropenic fever (3/11). Among the three cases of fever and neutropenia, a microbiologically defined focus could only be identified in two cases: a urinary tract infection caused by ESBL-producing *E. coli* and an infection by *Clostridioides difficile*. Of the hemato-oncologic patients who developed infectious complications and were colonized by GNB, only in 2 cases (18.2%) the same organisms were isolated in blood cultures as in oral cavity swabs. Bacteremia due to ESBL-producing *E. coli* and MDR *Pseudomonas* spp. was reported in these patients, respectively.

During the follow-up period, four (11%) colonized hemato-oncologic patients and two (2.9%) non-colonized hemato-oncologic patients died (OR, 4.06; 95% CI 0.70–23.36; P = 0.17). The causes of death among colonized hemato-oncologic patients were septic shock (3 deaths), and one death was attributed to the underlying disease. The two deaths reported in the non-colonized hemato-oncologic patients were due to stroke and septic shock, respectively.

## Discussion

In the present study, the rate of oral colonization by GNB was twice as high in patients with hematologic malignancies and solid tumors compared with healthy subjects (34% vs. 17%). This finding is consistent with a previous study reporting an oral colonization rate of 37% in patients with chronic lymphocytic leukemia [20]. There is evidence that oral colonization by GNB is a marker of clinical severity [21]. In this present work, GNB isolates were more common in subjects with a higher number of comorbidities evaluated by the Charlson scale. These results suggest an association between clinical severity and colonization by GNB.

On the other hand, healthy individuals are rarely carriers of GNB, with oral colonization rates ranging from 2 to 8% [20–22]; while in our study, we found a rate of 17%. This difference between the reported oral colonization rates in healthy subjects may be attributed to the heterogeneity of the isolation methods used and the transient nature of GNB colonization in this population [22].

Regarding colonization with clinically relevant bacteria considered in this study, a higher rate of these organisms was found in the hemato-oncologic patients’ group compared to healthy controls (14.5% vs. 4.8%). In the last few years, there has been a transition from the predominance of Gram-positive organisms to GNB as etiologic agents of infections in hemato-oncology patients [7]. In particular, an increased incidence of bacteremia due to *E. coli, Pseudomonas aeruginosa*, and *Acinetobacter baumannii* has been reported [23]. The higher proportion of colonization by these bacteria is significant as they often possess intrinsic resistance mechanisms and are frequently acquired in hospital settings. Colonization by these bacteria is associated with a worse prognosis and increased risk of subsequent infections [8, 14, 15].

Previous studies have shown that maintaining good oral hygiene and moist oral mucosa is closely associated with a reduced risk of colonization and infection by pathogenic microorganisms in hematologic patients [4]. However, the oral hygiene of hospitalized patients is compromised by the disease and the unfamiliar environment, which hinders adherence to regular dental hygiene practices. In our study, it was observed that the group of hemato-oncologic patients exhibited lower adherence to hygienic-dietary habits and a higher colonization rate. Consensus and protocols for dental care have been established to prevent oral complications, especially for patients undergoing hematopoietic stem cell transplantation [24]. However, the recommended approach remains controversial in other cancer patients undergoing chemotherapy [25].

Before and during cancer treatment, intensive oral care reduces the risk of oral complications associated with chemotherapy, which promotes bacterial adhesion and colonization [25]. The efficacy of oral rinses in eradicating oral GNBs in cancer patients remains unclear because no specific preparation shows superiority [26, 27]. However, the present study found that using mouth rinses and frequent dental visits were associated with a low rate of GNB colonization. Therefore, we emphasize the necessity of reinforcing patient education on self-care for dental health as a strategy to prevent oral colonization by GNB.

Another proposed strategy to eradicate colonizing organisms in hemato-oncologic patients is empiric antibiotic therapy and gastrointestinal decolonization. Currently, there is limited evidence to recommend these strategies due to the alteration of the gut microbiota, which includes the selection of resistant bacteria; in fact, they could constitute a factor associated with colonization and bacteremia by resistant pathogens [9, 28, 29]. In our study, antibiotic therapy was one of the associated factors with oral colonization by third-generation cephalosporin-resistant GNB. Additionally, we found that oncology patients with higher functionality as measured by the ECOG scale (0–2) were less frequently colonized by resistant GNB. In contrast, hemato-oncologic patients with a higher Charlson comorbidity scale were more frequently colonized with resistant GNB. These results suggest an association between patients’ functional status, clinical severity, and colonization with resistant GNB. To our knowledge, this is the first report showing that the optimal functional status of patients is a protective factor for colonization with resistant GNB.

An additional recognized risk factor for GNB colonization is the duration of severe neutropenia [30]. However, our study did not demonstrate a significant association with oral colonization, this result may be due to the fact that we only evaluated the absolute neutrophil count. Regarding chemotherapy, we hypothesize that the lack of significant association with oral colonization may be attributed to the heterogeneity of chemotherapy regimens administered in the oncology patients’ group, as the myelosuppressive potential varies according to the dose and type of chemotherapeutic agent, as well as the duration of the regimen [6, 31]. Prospective, observational studies are needed to better understand chemotherapy’s contribution to oral colonization and its potential consequences.

One of the strengths of the present study is the prospective 30-day evaluation of the patients’ clinical outcomes. Infectious complications occurred more frequently in patients with oropharyngeal isolation of GNB compared to the non-colonized group (30.5% vs. 2.9%). In two (18%) of 11 patients who developed infectious complications, the same bacterial genus was isolated from both oral swabs and blood cultures during hospitalization (*E. coli* and *Pseudomonas* spp.). Similar results were reported by Cattaneo et al. [9] in their six-month follow-up study, where 25.7% of GNB MDR colonized patients developed bacteremia, 16% of which were attributed to the same pathogen, and 11.8% were unrelated to the pathogen. However, our study is limited by the lack of molecular biology techniques to confirm whether the bacterial species colonizing the oral cavity were genotypically identical to those causing infectious complications.

Regarding mortality, a higher rate was observed in colonized oncology patients compared to non-colonized patients (11% vs. 2.9%). The lack of statistically significant differences may be due to the size of the subgroup. Other authors have reported that carrying GNB MDR is an independent predictor of mortality even after hospital discharge [8].

This study has several limitations. First, this was a single-center study, which may limit the generalizability of the findings. Second, molecular biology techniques were not employed to confirm the genotypic similarity between the bacterial species colonizing the oral cavity and those causing infectious complications. Another limitation was that participants’ education level and socioeconomic status were not included as variables in the analysis, which could have influenced their hygienic and dental hygiene practices.

## Conclusion

In conclusion, the rate of oral colonization by GNB was higher in patients with hematologic malignancies and solid tumors compared to healthy subjects. Also, the colonized patients with GNB were associated with higher rates of infectious complications. There is a knowledge gap regarding dental hygiene practices in oncology patients colonized by GNB. Our results suggest that patients’ hygienic-dietary habits, especially frequent dental visits, are a protective factor against colonization. Therefore, we consider that the first step to preventing oral colonization in oncology patients should be based on the promotion of dental hygiene and maybe the early identification of patients with GNB colonization, particularly those with resistant strains. Future research should be focused on determining the effectiveness of dental hygiene practices as well as other strategies to reduce and eradicate oral colonization by GNB in cancer patients.

## Electronic supplementary material

Below is the link to the electronic supplementary material.

Supplementary Material 1

## Data Availability

The datasets used and/or analyzed during the current study are available from the corresponding author on reasonable request.

## List of abbreviations

GNB: Gram-negative bacilli
MDR: multidrug-resistant
ESBL: Extended-spectrum beta-lactamase
SD: standard deviations
IQR: interquartile ranges
OR: odds ratio
