# Supplementary material for: Oral colonization by gram-negative bacilli in patients with hematologic malignancies and solid tumors compared with healthy controls
Source: BMC Oral Health. 2023 Jul 8;23:465. doi: 10.1186/s12903-023-03172-y (PMC10329337; doi:10.1186/s12903-023-03172-y)
Supplement: Supplementary file 1 — Supplementary Material 1 [file 12903_2023_3172_MOESM1_ESM.docx]

**Supplementary material**

| Supplementary Table 1. Analysis of factors associated with oral colonization by third-generation cephalosporin-resistant GNB in hemato-oncologic colonized patients. | | | | |
| --- | --- | --- | --- | --- |
| Characteristic | **Colonized by third-generation cephalosporin-resistant GNB**  **(N=12)**  **n (%)** | **Colonized by third-generation cephalosporin-sensitive GNB**  **(N=24)**  **n (%)** | **OR**  **95% CI** | **P value** |
| Diagnosis  Acute leukemias  Lymphomas  Multiple Myeloma  Solid tumor | 1 (8.3)  2 (16)  0  9 (75) | 5 (20)  6 (25)  1 (4.2)  12 (50) | 0.34 (0.03-3.35)  0.6 (0.10-3.54)  -  3 (0.64-13.88) | 0.63†  0.69 †  1.0 †  0.28* |
| Prolonged hospital stay (>21 days) | 2 (16) | 1 (4.2) | 4.6 (0.37-56.75) | 0.53 † |
| Antibiotic therapy | 8 (67) | 6 (25) | 6 (1.32-27.28) | **0.028** † |
| ECOG  0-2  3-5 | 1 (8.3)  11 (92) | 16 (66)  8 (33) | 0.04 (0.005-0.41) | **0.001*** |
| Charlson  Low risk (0-2)  Intermediate risk (3-4)  High risk (≥5) | 2 (16)  3 (25)  7 (58) | 11 (45)  8 (33)  5 (20) | 0.23 (0.04-1.31)  0.66 (0.14-3.16)  5.32 (1.17-24.14) | 0.14 †  0.71 †  **0.031** † |
| Neutropenia | 2 (16) | 6 (25) | 0.6 (0.10-3.54) | 0.69 † |
| Severe neutropenia (<500 cells/µL) | 1 (8.3) | 2 (8.3) | 1 (0.08-12.27) | 1.0 † |
| Lymphopenia (<1000 cells/µL) | 4 (33) | 9 (37) | 0.83 (0.19-3.57) | 1.0 † |
| History of previous hospitalizations | 9 (75) | 13 (54) | 2.53 (0.54-11.76) | 0.29 † |
| Recent chemotherapy | 4 (33) | 13 (54) | 0.42 (0.09-1.79) | 0.40 * |
|  |  |  |  |  |

GNB: Gram-negative bacilli. OR: Odds ratio.

* Chi-square

† Fisher's exact test
